# Knowing where the nose is

**DOI:** 10.1186/s12915-017-0382-6

**Published:** 2017-05-15

**Authors:** Winthrop F. Gillis, Sandeep R. Datta

**Affiliations:** Department of Neurobiology, Harvard Medical School, Boston, MA 02115 USA

## Abstract

Improvements in imaging technology and the development of powerful machine learning algorithms are revolutionizing the study of animal behavior in the laboratory. These innovations promise to reveal both global and local features of action relevant to understanding how the brain functions. A study in *BMC Biology* describes one such tool called OptiMouse, which is an open source platform that uses video to capture key features of mouse behavior, including information relevant to olfactory investigation.

See research article: 10.1186/s12915-017-0377-3

## Commentary

Traditionally, studies of rodent behavior have been performed using focused lenses: for conceptual and/or technical reasons high-dimensional behavior is collapsed into a much smaller number of dimensions, which are usually hand-selected based upon the hypothesis the researcher wishes to test [1]. For example, the locomotory exploration of an open field by a mouse—a simple yet incredibly rich pattern of behavior—is typically reduced into a single metric capturing the number of times the mouse enters the center of the arena [2]. The recent availability of cheap and high-resolution video cameras, powerful computing hardware, and sophisticated statistical techniques, adapted from fields such as machine vision and machine learning, is enabling a dramatic shift towards more quantitative and objective methods of behavioral analysis. Significant improvements have been made over the past 5 years in camera resolution, feature extraction, animal tracking, supervised behavioral identification, and unsupervised identification of behavioral modes or motifs, for example, and many of these methods have been packaged into end-to-end pipelines in which rodents are imaged on the front end and a dizzying array of parameters describing behavior are spit out the back end [3–6].

However, these pipelines have two important constraints that often limit their usefulness. First, there is limited generalization. The code that converts video images into data relies upon a set of parameters that typically are specific to a particular camera, lighting condition, and arena. If these parameters are hard-coded (as they often are), analysis of video obtained under experimental conditions that differ from those used to build the analytical pipeline can fail, limiting the types of hypotheses one can test. Second, there is a lack of transparency: often these parameters are hidden so far under the hood that it is not clear why some videos are easily handled while others remain refractory to analysis. Addressing these problems is crucial given the parallel advances being made in methods for probing the structure and function of the nervous system, including gene editing, pharmaco- and optogenetics, and high-density neural recordings; understanding how manipulating a gene or neural circuit influences behavior—or how patterns of neural activity might be correlated with patterns of action—will necessarily require the generation and analysis of large-scale behavioral data, largely in the form of video.

**Fig. 1:**
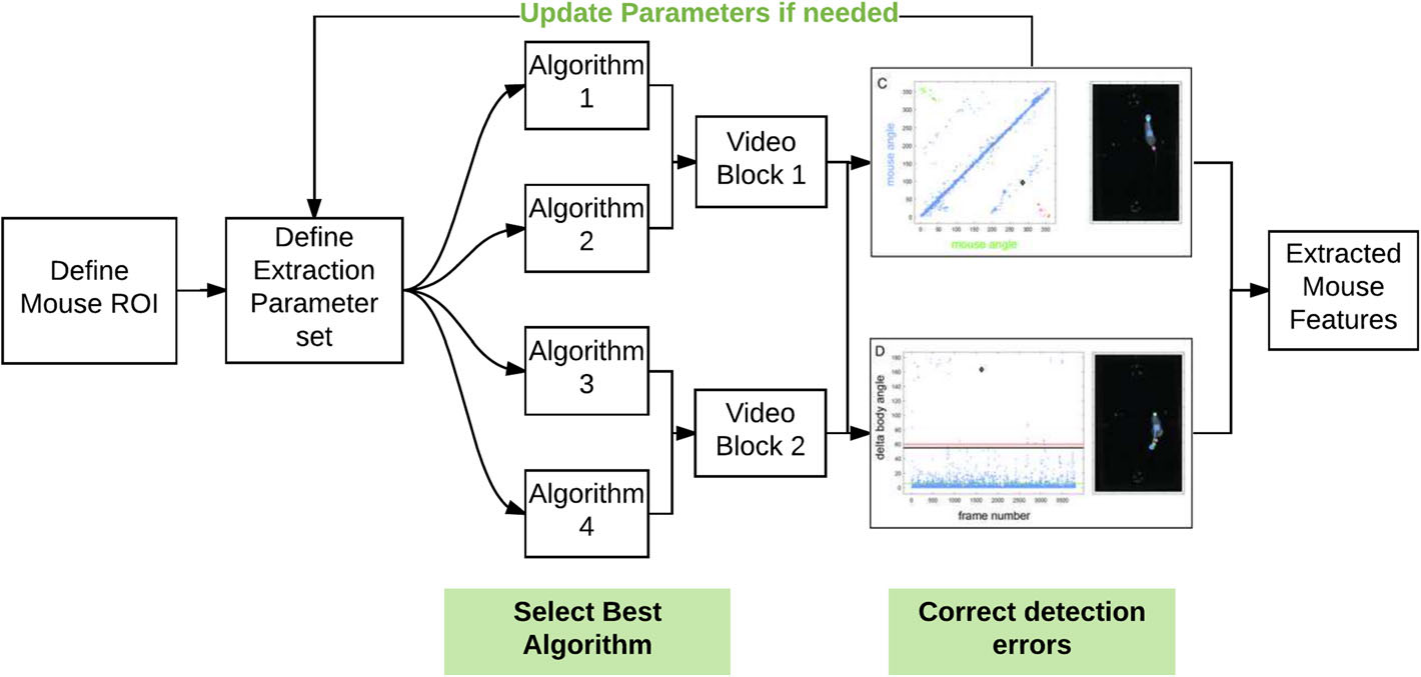
OptiMouse enables a configurable and interactive workflow to minimize mouse feature errors within a video recording. Multiple parameter sets can be defined for mouse extraction (*left panels*), then the best detection algorithm is picked to minimize detection errors (*middle panels*). Different parameters and algorithms can be used on different blocks of the same video for optimal detection. The whole process can be repeated until the desired detection performance is reached (*right panels*). Figure adapted from [7]. *ROI* Region of interest

Work published this month in *BMC Biology* from Yoram Ben-Shaul and colleagues adds significantly to the field by providing an open-source MATLAB-based toolkit (called OptiMouse) to identify a mouse’s spatial features within a given experiment [7]. Although there are available methods that provide some of its functionality, OptiMouse is explicitly built for configurability, and hence generalization. Users input video into the pipeline, and then are able to toggle through different sets of parameters to observe how each affects the extraction process, eliminating errors for any measured variable (like the mouse centroid, or the position of the nose; Fig. 1). If the multiple image processing algorithms that are supplied with OptiMouse are unsuitable, the user can supply his own algorithm for mouse detection. OptiMouse allows explicit comparisons to be made between parameter sets and addresses non-stationarities in the video (a common problem in real-world experimental scenarios) by enabling distinct parameter sets to be applied to different segments of any behavioral video. This capacity is particularly powerful, as in effect OptiMouse represents not one solution to the problem of behavioral image analysis, but many. Coupled to this is the ability to quickly review extracted data to get a sense of the number and nature of errors that remain after applying the parameter sets, enabling fast correction. Thus, human supervision plays an essential role in using OptiMouse, but the “pain” of reviewing videos is minimized with rational interface design and automation.

In addition to developing a platform for rapid and transparent behavioral analysis, OptiMouse explicitly measures the position of the mouse’s nose with respect to the mouse’s body and arena. Olfaction is an essential sense used to forage for food in the wild, to avoid potentially deadly conflicts with conspecifics or predators, and to obtain suitable mates [5]. Because olfaction is an active sense—optimal sensory interrogation requires the nose to be actively positioned by the mouse, followed by rapid inhalation to facilitate odor sampling—understanding the position of a mouse’s nose is crucial for understanding how the mouse processes odor information [8]. Indeed, a large subset of the mouse’s behavior in a given arena appears to be some sort of rearing or sniffing behavior, as if their body dynamics are disproportionately devoted to probing the olfactory world [9]. However, nose tracking is notoriously difficult for most automated behavioral classification software, in part because the context in which the nose is found in the video is constantly evolving. As a consequence of this limitation, we lack an understanding of both how basic odor sampling is accomplished by rodents and how neural activity in olfactory centers might be altered as a consequence of active sampling.

**Fig. 2:**
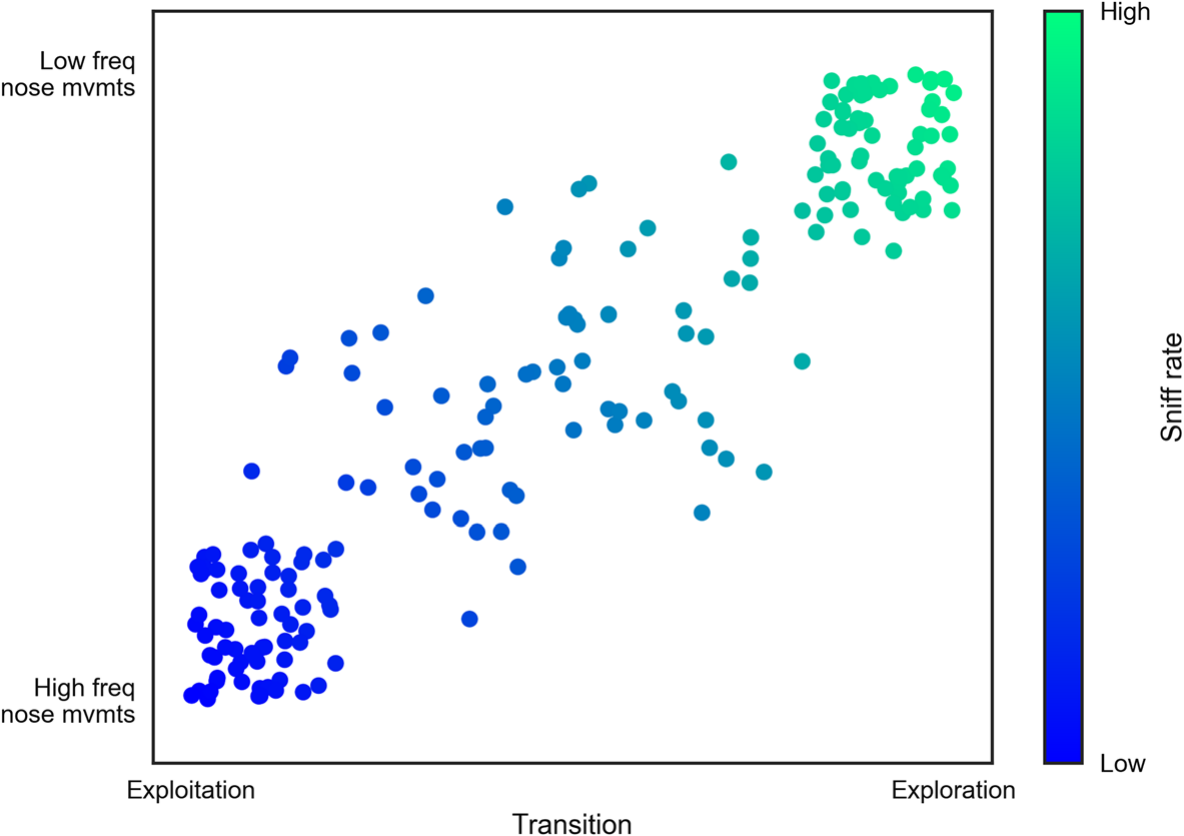
A cartoon dataset illustrating how measuring where the nose is may lead to insight into the underlying strategies used by mice to explore and exploit resources in their environment. In this cartoon, high frequency nose movements reflect investigatory strategies associated with local exploitation, whereas low frequency movements are used to interrogate space as part of exploratory strategies

The development of OptiMouse therefore raises the possibility of building assays in the lab explicitly designed to probe different strategies for olfactory sampling. For example, foraging animals could engage in exploratory or exploitative strategies depending upon internal and external factors [10]. By better defining the dynamics of nose position in relation to odor objects in an arena, OptiMouse may be useful for revealing how neural representations for odors are modulated as a function of position, and lead to sensible behavioral decisions in response to a given cue. For instance, one could imagine that sweeping low frequency head movements employed during exploration are optimized to sample a broader region of space, in order to sample a novel environment more quickly, while short high frequency head movements employed during exploitation are optimized to facilitate short travel times to a known target such as a food patch (Fig. 2).

Given the robustness of the image processing framework within OptiMouse, one could even imagine using this tool in the future to explore olfactory sampling in complex environments such as those including multiple mice. By design, OptiMouse is meant to be modular, and can integrate with Matlab data processing code seamlessly, allowing it to be updated by a user community over time. While machine vision approaches to characterizing behaviors are currently challenged by complex or dynamic environments, as tools for segmenting objects in video data improve, the capability of OptiMouse can be augmented to enable ever-more sophisticated measurements of mouse behavior.
